# The Importance of Sleep for People With Chronic Pain: Current Insights and Evidence

**DOI:** 10.1002/jbm4.10658

**Published:** 2022-06-17

**Authors:** Katie Whale, Rachael Gooberman‐Hill

**Affiliations:** ^1^ Bristol Medical School University of Bristol Bristol UK; ^2^ National Institute for Health Research Bristol Biomedical Research Centre University Hospitals Bristol and Weston NHS Foundation Trust and the University of Bristol Bristol UK

**Keywords:** PRACTICE/POLICY‐RELATED ISSUES, DISEASES AND DISORDERS OF/RELATED TO BONE, EPIDEMIOLOGY, ORTHOPAEDICS

## Abstract

We are currently in the midst of a sleep crisis. Our current work and lifestyle environments are normalizing poor sleep with substantial negative impact on our health. Research on sleep has linked sleep deprivation to poorer mental health, obesity, cancer, diabetes, heart disease, and a myriad of other health conditions. Sleep deprivation is an even greater issues for people with musculoskeletal conditions and chronic pain. Between 67% and 88% of individuals with chronic pain experience sleep disruption and insomnia, and at least 50% of people with insomnia report chronic pain. The link between sleep and pain is well documented. Experimental, cohort, and longitudinal studies have all demonstrated that restricted sleep is linked to greater pain. Poor sleep therefore not only affects general health but has a direct impact on inflammation, pain response, and experience. Improving sleep in people living with musculoskeletal conditions and with chronic pain has the potential to deliver great benefit to many. This article describes the evidence base that can underpin such work, including research about the link between pain and sleep as well as theories and approaches to intervention that may help. © 2022 The Authors. *JBMR Plus* published by Wiley Periodicals LLC on behalf of American Society for Bone and Mineral Research.

## Introduction

Good quality sleep is essential to health and wellbeing across the whole life course. Sleep deprivation is associated with mental health difficulties,^(^ ^1^ ^)^ obesity,^(^ ^2^, ^3^ ^)^ cancer,^(^ ^4^, ^5^ ^)^ type 2 diabetes,^(^ ^6^ ^)^ heart disease,^(^ ^7^ ^)^ and many other health conditions. Conversely, good quality sleep supports physical recovery, memory consolidation, learning, and positive mood.^(^ ^8^ ^)^ Poor sleep is common among people living with painful musculoskeletal conditions and can have a serious negative impact on their lives and pain management. Addressing sleep and providing ways to support and improve sleep can provide benefit to many. We suggest that there is a clear need to develop, evaluate, and implement care for sleep among people living with musculoskeletal pain. This article describes the evidence base that can underpin such work, including research about the link between pain and sleep as well as theories and approaches to intervention that may help.

## Prevalence of Sleep Issues and Chronic Pain

Sleep deprivation and interrupted sleep are substantial issues for people who experience chronic pain (pain lasting longer than 3 months). A recent systematic review on the prevalence of sleep disturbance for those with non‐cancer pain indicates that between 72% and 75% of this population experience sleep disturbance,^(^ ^9^ ^)^ with other research putting the figure at 88%.^(^ ^10^, ^11^ ^)^ Musculoskeletal conditions are frequently associated with sleep issues with prevalence of up to 65% in rheumatoid arthritis, 70% in osteoarthritis, and 95% in fibromyalgia.^(^ ^9^ ^)^ Individuals who experience both chronic pain and sleep problems are likely to have greater pain severity, longer duration of pain, greater disability, and be less physically active than those without sleep disturbance.^(^ ^12^ ^)^ In addition, people who have both pain and sleep difficulties are more likely to experience depression, catastrophizing, anxiety, and suicide ideation.^(^ ^12^ ^)^

## Link Between Sleep and Pain

There is a robust evidence base for the link between sleep and pain. Experimental, cohort, and longitudinal studies have all demonstrated that restricted sleep is linked to greater pain. Experimental studies have examined the short‐term impact of sleep restriction on pain, commonly using pain threshold tests such as cold pressure. These studies have consistently shown that sleep deprivation in healthy subjects, in particular slow wave sleep restriction (deep restorative sleep), is associated with increased pain stimulus responses.^(^ ^10^, ^13^ ^)^ However, these approaches have limited generalizability for people with chronic pain because they do not mirror their experience. People living with chronic pain commonly experience waking several times each night as well as long‐term reduced sleep quality. Some studies have sought to address this by using “forced awakening” techniques, which forcibly awaken participants multiple times per night. Smith and colleagues^(^ ^14^ ^)^ conducted a study in which otherwise healthy women were awakened at eight intervals during the night over an 8‐hour sleep period. This restricted their total sleep time to 280 minutes (just over 4.5 hours). Compared with a group who had restricted sleep (same total sleep time but uninterrupted) and a control group who slept for 8 hours, forced awakening was associated with greater next‐day spontaneous pain reports and reduced conditioned pain modulation (reduction in the body's ability to process pain resulting in greater pain experiences).

Prospective longitudinal studies focusing on the effect of sleep on future pain have reported similar findings. Studies in people who experience headaches and migraines have shown that elevated insomnia symptoms increase the risk of exacerbating existing headache, and in developing new headache symptoms at long‐term follow‐up ranging from 1 to 12 years.^(^ ^15^, ^16^ ^)^ Sleep quality has also been examined in relation to postsurgical pain: preoperative sleep quality affects postoperative pain,^(^ ^17^, ^18^, ^19^ ^)^ including joint arthroplasty.^(^ ^20^ ^)^ This is of particular interest in chronic pain research as joint replacements are predominantly carried out to relieve the symptoms of chronic pain for conditions such as osteoarthritis.

## Temporal Relationship Between Sleep and Pain

A subject of recent research has been the temporal relationship between sleep and pain and the day‐to‐day predictive associations. The bidirectionality of the relationship is widely accepted,^(^ ^10^, ^21^ ^)^ with poor sleep leading to worse pain and pain negatively impacting sleep; however, the strength and direction of the association is less clear. There is growing body of evidence that suggests a temporal precedence for sleep over pain, such that sleep impairment is a stronger predictor of pain than pain is a predictor of sleep impairment.^(^ ^12^, ^13^ ^)^ A study including adolescents with a range of chronic pain conditions found that total sleep time and wake after sleep onset (waking during the night) were associated with next‐day pain reports; however, pain levels did not predict sleep quality or efficiency.^(^ ^22^ ^)^

Sleep problems have been identified as a risk factor for development of musculoskeletal pain. A Swedish prospective population study identified that problems with initiating sleep, maintaining sleep, early awakening, and nonrestorative sleep predicted the onset on chronic widespread pain over 5 and 18 years in individuals with no pain at baseline, irrespective of mental health status. In addition, sleep problems and fatigue independently predicted chronic widespread pain at 5 years.^(^ ^23^ ^)^ Research has suggested the underlying mechanism for this association is increased systemic inflammation.^(^ ^24^ ^)^ New research examining this relationship has found that this association is mediated by high or low affect (mood/emotional state).^(^ ^25^ ^)^ Low positive affect and sleep disturbance were associated with increased inflammation levels, and high positive affect identified as a protective factor.

## Relevance for Research and Treatment of Chronic Pain

Musculoskeletal chronic pain conditions come with different pain profiles, and sleep experience may vary according to condition. Understanding the nature of the relationship between sleep and pain in a variety of conditions may provide key information for design of treatment approaches.

As well as defining pain by reference to condition or diagnosis, considering pain type without reference to associated condition provides key information that may be relevant to sleep. Nociceptive and inflammatory pain is associated with damage to tissue, such as osteoarthritic joint damage.^(^ ^26^ ^)^ Nociceptive pain (pain caused by damage to body tissue) is commonly treated with traditional analgesics and anti‐inflammatory medication.^(^ ^27^ ^)^ Neuropathic pain is associated with changes to the nerves themselves and affects the way pain signals are sent back to the brain.^(^ ^28^ ^)^ Medicines that may provide benefit for people with nociceptive pain may do little to alleviate neuropathic pain symptoms. Of the 20% of the population who live with chronic pain in the UK, approximately 8%–9% experience chronic neuropathic pain,^(^ ^29^ ^)^ highlighting a large population who may not benefit from conventional pharmacological pain management.

In 2017, a new category of pain experience was introduced by the International Association for the Study of Pain (IASP): “nociplastic” pain.^(^ ^30^ ^)^ Nociplastic pain is defined as “pain arising from the altered function of pain‐related sensory pathways in the periphery or central nervous system, causing increased sensitivity.”^(^ ^31^ ^)^ This type of pain can occur in isolation or alongside chronic pain conditions that are primarily nociceptive or neuropathic. Nociplastic pain in common in fibromyalgia and is thought in part to be due to changes in how pain is processed by the nervous system, such as in central sensitization (increased pain response/pain hypersensitivity to external stimuli).^(^ ^32^ ^)^

Nonpharmacological treatment approaches focused on pain management are the first line recommendation for nociplastic pain, and these include sleep hygiene (healthy sleep habits). Along with patients who experience neuropathic pain, sleep interventions may offer a positive treatment approach for nociplastic pain.

## Intervention Approaches

Interventions to improve sleep for people with pain include pharmacological and a range of other approaches. Although pharmacotherapy treatments may offer short‐term solutions to problems such as sleep latency (taking a long time to fall asleep), they may have unwelcome side effects and are not recommended for long‐term use.^(^ ^33^ ^)^ Behavioral and psychological interventions have gained traction in recent years as ways to improve sleep without side effects and to provide long‐term support.

Our recent systematic review of nonpharmacological sleep interventions for chronic pain identified a large range of existing sleep interventions including relaxation, mindfulness, physical therapies, and exercise.^(^ ^34^ ^)^ Cognitive behavioral therapy (CBT) approaches provided the largest evidence base, and these included CBT for insomnia (CBT‐i), CBT for pain (CBT‐P), and combined approaches (CBT‐iP). CBT‐i can be delivered on an individual or group basis and consists of a course of sessions focusing on psychoeducation and sleep hygiene information, sleep restriction, relaxation, stimulus control, and cognitive therapy.

Evidence about the effectiveness of CBT for improving sleep indicates that CBT can provide equal benefit or be superior to pharmacotherapy.^(^ ^35^ ^)^ Systematic reviews of CBT interventions demonstrate significant improvements in sleep quality in the short and medium term for CBT‐i^(^ ^34^ ^)^ and for global measures of sleep.^(^ ^36^ ^)^ Condition specific reviews including patients with lower back pain, fibromyalgia, and osteoarthritis show similar results with CBT therapies improving short‐term sleep outcomes.^(^ ^36^, ^37^, ^38^, ^39^ ^)^

CBT may be particularly suitable for people with chronic pain because such approaches can address pain and sleep in tandem. Some individuals who live with chronic pain may engage in “pain catastrophizing.” Individuals who experience pain catastrophizing experience greater pain related fear (fear of physical movement and activity resulting in pain), this can lead to pain avoidant behaviors and pain hypervigilance.^(^ ^40^ ^)^ Engaging in the fear‐avoidance cycle of pain means it can be very difficult for these individuals to focus on anything other than their pain or break this cycle 66–67. Pain catastrophizing has an additional negative impact on pain related sleep issues as pain rumination contributes to sleep disturbance.^(^ ^41^, ^42^ ^)^ CBT‐P and CBT‐iP have been shown to improve pain coping, reduce catastrophizing, and increase pain acceptance.^(^ ^43^ ^)^

Until recently, behavioral and psychological therapies were usually delivered in person either on a one‐to‐one or group basis. Increasingly, therapies are delivered remotely using video appointments, websites, or digital apps. Websites and apps may deliver automated CBT, and studies that have evaluated such approaches have found them to be an effective and acceptable means of delivery.^(^ ^44^, ^45^ ^)^ With growth in the online wellness industry, the range of smartphone apps providing digital access to relaxation and mindfulness methods has increased substantially in recent years. Unlike the evidence base for automated CBT, evidence relating to relaxation and mindfulness is less developed; however, a studies of a commercial relaxation app found that most users reported improved sleep, including falling asleep and staying asleep, and overall sleep quality.^(^ ^46^, ^47^ ^)^ However, findings were limited to a sample who were primarily female and who had high levels of education. Socioeconomic factors are an important consideration when designing and assessing the impact of digital sleep interventions; although digitally enabled interventions may provide an accessible route for many, those without digital access may be excluded. Availability of devices, digital literacy, internet access in rural and urban areas, and the range of language availability need to be considered.^(^ ^48^ ^)^

## Support for Change: The Role of Behavior Change Theories

Individuals' beliefs about their health conditions or experiences can have considerable impact on engagement in interventions—such as CBT—that require behavior change. From health psychology, the common sense model of health representation, first proposed by Leventhal, Meyer, and Nerenz, focuses on the relationship between illness‐representation (individual beliefs and expectations about an illness), coping, and health outcomes.^(^ ^49^ ^)^ This model suggests that perceived causes of a condition and the curability or controllability form part of an individual's illness perception. This perception then impacts how someone responds to treatment recommendation. Although musculoskeletal conditions may be associated with different types of sleep difficulties, it is also likely that perceived causes of a condition, curability, and controllability weigh heavily in beliefs about sleep. Furthermore, in current society, although sleep is increasingly the subject of wellness intervention, poor sleep (particularly short duration of sleep) is frequently normalized or accepted as part of life.^(^ ^50^ ^)^ People who live with painful musculoskeletal conditions may see poor sleep as an inevitable consequence of living with pain^(^ ^51^ ^)^ and as out of their personal control.^(^ ^52^ ^)^ Addressing these deeply held views about sleep and condition may be an important element of methods to improve sleep.

Individuals with chronic pain may experience disturbed sleep for many months or years, which means that engagement with sleep interventions need to be long‐term. Despite an absence of evaluations of the longer‐term effectiveness of sleep interventions for people with chronic pain, health psychology offers guidance about how behavior change can be sustained. For instance, theories of motivation—such as self‐determination theory^(^ ^53^, ^54^ ^)^—posit that intrinsic motivation is key to long‐term change. Intrinsic motivation is internal personal motivation, which can be developed and supported through support for individuals' feelings of autonomy, competence, and relatedness. In other words, people are more likely to be motivated to change if they believe that they are in control of the change, feel able to achieve the change, and sense that they are supported by and connected to other people. Beliefs about sleep and pain may undermine feelings of autonomy and self‐efficacy. Reductions in these feelings may impact on motivation that would bolster and facilitate engagement in active treatments or behavioral change. Bringing focus on health beliefs and motivation together highlight the importance of education about sleep and pain alongside or within interventions that promote autonomous motivation and competence.

## Conclusions

Promoting good quality sleep is important for people with pain related to musculoskeletal conditions. A range of sleep problems can be addressed through existing interventional approaches that are underpinned by established theories. Identifying which approach to use when and with whom depends on a full understanding of individual health beliefs that relate to sleep as well as identification of barriers to behavior change. Progress in our understanding of the complex relationship between sleep and pain provides a promising basis for interventions that may improve sleep, help with pain, and augment health‐related quality of life. Future research to develop and evaluate tailored sleep interventions should identify whether support for sleep should be embedded into self‐management and healthcare provision.

## Author Contributions

**Katie Whale:** Conceptualization; formal analysis; writing – original draft; writing – review and editing. **Rachael Gooberman‐Hill:** Conceptualization; writing – review and editing.

### Peer Review

The peer review history for this article is available at https://publons.com/publon/10.1002/jbm4.10658.
